# Brittle Cornea Syndrome: Molecular Diagnosis and Management

**DOI:** 10.3390/diagnostics15131596

**Published:** 2025-06-24

**Authors:** Marco Zeppieri, Mattia Gentile, Antonio Acquaviva, Davide Scollo, Fabiana D’Esposito, Giuseppe Gagliano, Alessandro Avitabile, Caterina Gagliano, Lucia Lapenna

**Affiliations:** 1 Department of Ophthalmology, University Hospital of Udine, 33100 Udine, Italy; 2 Department of Medicine, Surgery and Health Sciences, University of Trieste, 34127 Trieste, Italy; 3 Department of Medical Genetics Di Venere Hospital, 70012 Bari, Italy; 4 U.O.C Ophthalmology, “Di Venere” Hospital, 70012 Bari, Italy; 5 Eye Clinic Catania University San Marco Hospital, Viale Carlo Azeglio Ciampi, 95121 Catania, Italy; 6 Imperial College Ophthalmic Research Group (ICORG) Unit, Imperial College, 153-173 Marylebone Rd, London NW15QH, UK; 7 Department of Neurosciences, Reproductive Sciences and Dentistry, University of Naples Federico II, Via Pansini 5, 80131 Napoli, Italy; 8 Department of Medicine and Surgery, University of Enna “Kore”, Piazza dell’Università, 94100 Enna, Italy; 9 Department of Ophthalmology, University of Catania, 95123 Catania, Italy; 10 Mediterranean Foundation “G.B. Morgagni”, 95125 Catania, Italy

**Keywords:** keratoglobus, genetic corneal disease, PRDM5 gene, irregular astigmatism, central corneal thinning, pachymetry

## Abstract

**Background and Clinical Significance:** Brittle cornea syndrome (BCS) is a rare, autosomal recessive connective tissue disorder characterized by extreme corneal thinning, high myopia, and increased risk of spontaneous or trauma-induced ocular rupture. It is primarily caused by mutations in the ZNF469 or PRDM5 genes, which regulate extracellular matrix integrity. Early recognition and diagnosis of BCS are crucial to prevent severe visual impairment. This report presents two genetically confirmed cases of BCS in Albanian siblings, emphasizing the diagnostic value of whole-exome sequencing and individualized surgical management strategies. **Case Presentation:** Two siblings—a 28-year-old male and a 25-year-old female—presented with progressive visual deterioration and marked corneal thinning (<200 µm). Both had a history of spontaneous ocular rupture following minor trauma in the contralateral eye. Detailed ophthalmologic evaluation revealed keratoglobus, high myopia, and irregular astigmatism. Genetic testing identified the homozygous pathogenic variant c.974delG (p.Cys325LeufsX2) in the PRDM5 gene in both cases. The male underwent penetrating keratoplasty (PKP), achieving a best-corrected visual acuity (BCVA) of 20/30. The female initially underwent deep anterior lamellar keratoplasty (DALK), which was converted to PKP intraoperatively due to central endothelial perforation, resulting in a BCVA of 20/25. Both patients remained complication-free over a 7-year follow-up period. **Conclusions:** These cases highlight the importance of early genetic diagnosis and a tailored surgical approach in managing BCS. Long-term monitoring and protective strategies are essential to prevent complications. Incorporating genetic testing into clinical practice can enhance diagnostic accuracy and guide personalized treatment plans in patients with hereditary corneal dystrophies.

## 1. Introduction

**Figure 1:**
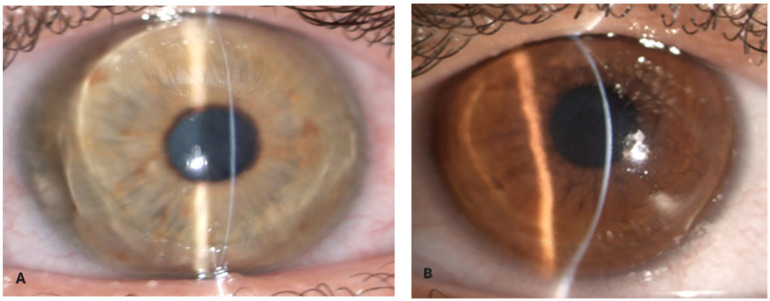
Ocular findings in a patient with brittle cornea syndrome. (A) Narrow slit-beam view of the right cornea with ectasia (Case 1), (**B**) (Case 2).

Brittle corneal syndrome (BCS) is a rare autosomal recessive connective tissue disorder that predominantly affects the cornea, leading to progressive thinning, increased fragility, and significant visual impairment [1,2]. The condition is linked to mutations in the ZNF469 and PRDM5 genes, both of which play essential roles in extracellular matrix homeostasis and collagen biosynthesis [3,4]. These genetic abnormalities lead to structural deficiencies that compromise corneal integrity, making affected individuals highly susceptible to spontaneous or trauma-induced corneal rupture [5,6]. BCS is often misdiagnosed due to its overlapping systemic manifestations, which include joint hypermobility (Figure 1), skeletal abnormalities, and sensorineural hearing loss. These features resemble those seen in other connective tissue disorders, particularly Ehlers–Danlos Syndrome (EDS), further complicating accurate diagnosis [1,7]. As a result, many patients do not receive appropriate clinical attention until severe ocular complications arise. The lack of wide-spread awareness and the limited literature on BCS contribute to diagnostic delays, underscoring the necessity of genetic testing in suspected cases [3,4,5].

From a clinical standpoint, BCS is marked by severe corneal thinning, with central corneal thickness (CCT) often measuring below 400 µm and, in extreme cases, even below 300 µm [3,4]. This severe thinning significantly weakens the structural integrity of the cornea, predisposing individuals to spontaneous perforation or rupture following minor trauma [4,8]. Patients may experience progressive myopia, astigmatism, and visual distortions, further affecting their quality of life [3]. Given the severity of these ocular manifestations, early detection and management are crucial to mitigate the risk of irreversible vision loss [3,5]. However, due to its rarity, BCS remains an under-recognized condition, often escaping early identification and intervention [2,6,9]. Raising awareness among ophthalmologists and geneticists is crucial for improving diagnostic rates and clinical outcomes [1,3,5].

In this study, we present two cases of genetically confirmed BCS in Albanian siblings, both of whom underwent keratoplasty.

We provide a detailed description of their clinical presentation, genetic findings, surgical outcomes, and long-term management strategies.

## 2. Detailed Case Description

**Figure 2:**
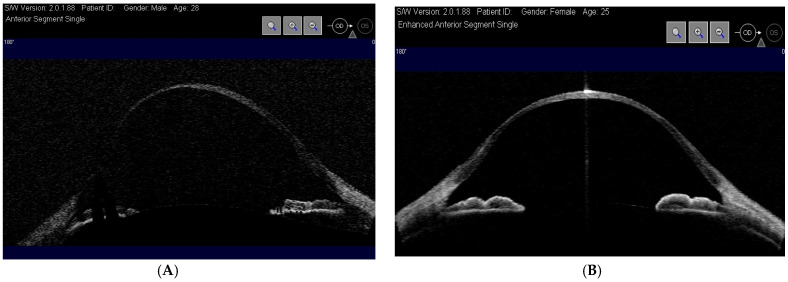
Optical coherence tomography (OCT-Visante) showing thin cornea right eye (Case 1) (**A**) and (Case 2) (**B**).

**Figure 3:**
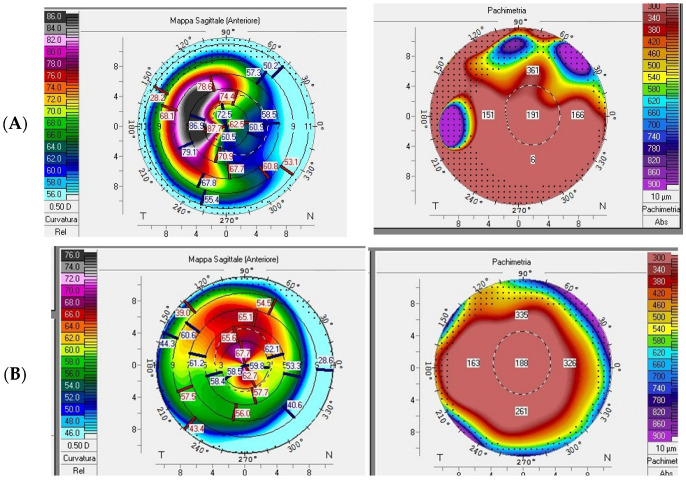
Pentacam refractive maps showing diffusely thin cornea on pachymetry map and abnormal keratometry values. (Case 1) (**A**) K-Max 88.6, AC depth 5.22 mm, thinnest point 151 µm; (Case 2) (**B**) K-Max 73, AC depth 3.84 mm, thinnest point 157 µm.

**Figure 4:**
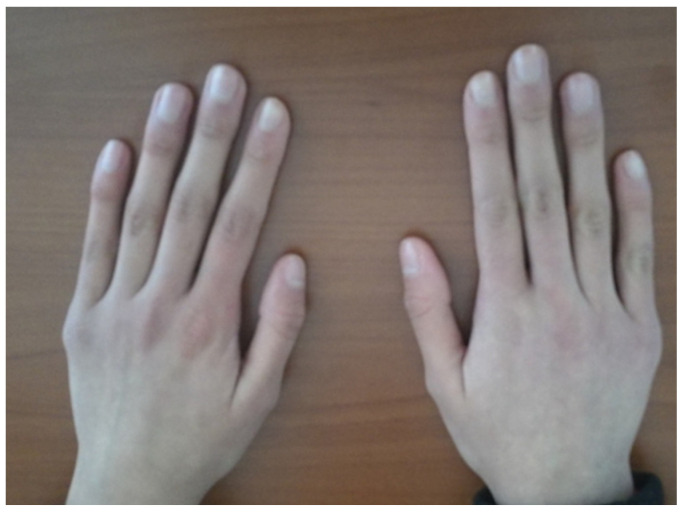
Hyperextensible metacarpophalangeal joint.

We evaluated two Albanian siblings affected by keratoglobus: a 28-year-old male (Case 1) and a 25-year-old female (Case 2). Both patients underwent corneal transplant after experiencing globe rupture in one eye following minor trauma and presented significant corneal thinning in the other eye [2,3]. Before surgery, both patients underwent a comprehensive ophthalmological evaluation, which included the following:

- Measurement of visual acuity: both best-corrected visual acuity (BCVA) and uncorrected visual acuity (UCVA);
- Slit-lamp examination: to assess corneal transparency, the presence of opacities, or other structural abnormalities (Figure 1);
- Corneal topography: to analyze corneal curvature and morphology (Figure 2);

- Corneal pachymetry: to measure corneal thickness at different points and determine the degree of thinning;
- Anterior segment optical coherence tomography (OCT): to obtain high-resolution images of the cornea and assess any structural alterations (Figure 3) [3];

- Systemic evaluation revealed sensorineural hearing loss and hyperextensible joints (Figure 4) [1,3];

- All examinations were performed both pre- and post-surgery to monitor the clinical progression of the patients after treatment.

This study was conducted in accordance with the principles of the Declaration of Helsinki. Both patients signed informed consent forms before starting the investigations. The diagnosis of brittle cornea syndrome (BCS) was confirmed through genetic testing [3,4,6,9]. Venous blood samples were collected from both patients, and whole-exome sequencing (WES) was performed on genomic DNA using the Ion AmpliSeq Exome platform (Thermo-Fisher, Waltham, MA, USA). Sequencing data were analyzed with platform-specific pipeline software, Torrent Suite v5.6 and Ion Reporter v5.1 [4,10]. From a therapeutic perspective, both patients underwent penetrating keratoplasty, a full-thickness corneal transplant (Figure 4), to restore visual function and reinforce corneal structure [2,3,8].

In this study, we describe two cases of genetically confirmed BCS in Albanian siblings, both of whom underwent keratoplasty (Table 1). In order to identify a genetic cause of familial clinical phenotype, whole-exome sequencing was performed. Homozygous variant c.974delG (p.Cys325LeufsX2) was identified in the PRDM5 gene, in both siblings. This variant was previously described in several cases and was classified as a pathogenic variant (Class 5) [3,5,6,9,10]. Both patients had a comprehensive systemic assessment. Alongside the ocular phenotype, sensorineural hearing loss and widespread joint hypermobility were seen. No dermatological anomalies or cardiovascular deformities were detected. Radiological assessment ruled out scoliosis and hip dysplasia.

### 2.1. Case 1: 28-Year-Old Male

A 28-year-old male presented with progressive visual deterioration, corneal thinning below 189 µm, and a history of recurrent corneal erosions [2,3]. Preoperatory evaluation revealed an axial length of 27.4 mm (right eye) and 27.2 mm (left eye); manifest refraction indicated myopia of 9.5D in right eye and 8.5 D in left eye; OCT assessed choroidal thickness as 220 µm. Given the severity of corneal thinning, the patient underwent penetrating keratoplasty (PKP). The graft was successful, and postoperative management included immunosuppressive therapy to prevent rejection. Over the follow-up period, the patient achieved a best-corrected visual acuity (BCVA) of 20/30 three months after keratoplasty. Continuous monitoring was required to assess the risk of potential complications, including graft rejection, secondary glaucoma, and corneal neovascularization [3,5].

### 2.2. Case 2: 25-Year-Old Female

The 25-year-old sister presented with similar symptoms but had a more pronounced corneal thinning, with a minimum corneal thickness of 157 µm [2,3,4]. Preoperatory evaluation revealed an axial length of 26.8 mm (right eye) and 27.1 mm (left eye); manifest refraction indicated myopia of 8.75D in right eye and 10D in left eye; OCT assessed choroidal thickness as 200 µm. To preserve corneal endothelium integrity and minimize complications, the patient initially underwent deep anterior lamellar keratoplasty (DALK). This approach initially resulted in better graft survival and fewer complications. However, during the surgery following a severe central endothelial perforation, the procedure had to be converted to penetrating keratoplasty (PKP). Postoperative recovery led to a BCVA of 20/25 approximately four months after keratoplasty, and the patient continued to be monitored for potential postoperative complications. The only recorded complication in both patients was the rupture of some individual suture stitches due to hydrolysis about two years after the procedure, causing minimal variations in astigmatism. The semi-annual follow-up examinations to which the patients are subjected have not detected any significant changes in visual acuity. Post-keratoplasty, the following therapeutic protocol was administered in both patients:

- Topical antibiotics (e.g., Moxifloxacin 0.5%) four times a day for 2 weeks.
- Topical corticosteroids (e.g., Fluorometholone 0.1% or Prednisolone 1%) initially four times a day, gradually reducing the dosage as directed by the ophthalmologist for 12 months.

Genetic findings and long-term follow-up genetic testing confirmed mutations in the PRDM5 gene, supporting the diagnosis of BCS in both cases [3,5,6,9,10]. The parents and close family members refused genetic testing but indicated no history of ocular or systemic connective tissue problems, except for the mother who was genetically analyzed, but no significant mutations were found. No other relatives were accessible for examination. The seven-year follow-up has shown no significant complications in either patient, highlighting the importance of individualized treatment strategies and long-term care to optimize visual outcomes.

### 2.3. Clinical Implications

These cases emphasize the need for the following:

- Early recognition of BCS and genetic testing to confirm diagnosis [1,3,7].
- Personalized surgical approaches, including DALK for better graft survival when feasible [3,4].
- Long-term monitoring to prevent and manage complications such as graft rejection and secondary glaucoma [3,5]. Beyond its clinical significance, the documentation of these rare BCS cases contributes to a broader understanding of the disease, helping to refine diagnostic criteria and treatment protocols. Future research should focus on genotype–phenotype correlations, novel therapeutic interventions, and strategies to enhance surgical success rates for patients with BCS [3,7].

## 3. Discussion

Brittle cornea syndrome (BCS) is a connective tissue disorder primarily affecting the eyes, with additional systemic manifestations such as developmental dysplasia of the hip, hypermobility of small joints, and sensorineural hearing loss. Due to its clinical similarities, BCS is often misdiagnosed as Ehlers–Danlos Syndrome [7]. Corneal topography in BCS typically reveals a diffusely thinned cornea, with a central corneal thickness (CCT) often below 400 µm and, in some cases, even less than 300 µm [3]. While corneal thinning occurs in other connective tissue disorders, the extent of thinning in BCS is notably more severe [3]. A definitive diagnosis and differentiation from other connective tissue disorders can be established through genetic testing, which identifies mutations in the ZNF469 gene (Type 1) and PRDM5 gene (Type 2) [3,4,5,6,9,10].

BCS is significantly underdiagnosed and often goes unnoticed until ocular trauma occurs or spontaneous corneal rupture develops, leading to a poor visual prognosis [8]. In our cases, genetic diagnosis played a pivotal role in confirming BCS, enabling early and targeted management. Given the rarity of the disease, early clinical suspicion is essential for timely genetic testing and appropriate intervention [3,4]. The identification of pathogenic mutations not only provides diagnostic confirmation but also serves as valuable information for genetic counseling and family planning [3,4].

Keratoplasty remains the primary treatment for BCS, as no pharmacological therapy has proven effective in slowing disease progression [3,9]. In both patients, penetrating keratoplasty resulted in significant visual improvement, highlighting the effectiveness of surgical intervention in hereditary corneal dystrophies. However, long-term success depends on factors such as graft survival, postoperative complications, and the risk of rejection, necessitating close postoperative monitoring and management [3,9]. These cases also underscore the importance of a multidisciplinary approach, involving ophthalmologists, geneticists, and corneal surgeons [3,9].

Collaboration among these specialists ensures comprehensive diagnostic evaluation and optimal therapeutic planning. Additionally, documenting such cases in the literature helps improve the understanding of BCS and may contribute to the development of standardized management protocols for future cases [2,3]. Further research is required to elucidate the molecular mechanisms underlying BCS and explore potential gene-targeted therapies [3]. Longitudinal studies evaluating the long-term outcomes of keratoplasty in BCS patients will provide insights into the durability and overall effectiveness of surgical intervention [9].

While the ocular manifestations are the most prominent features, systemic abnormalities, such as developmental dysplasia of the hip, hypermobility of small joints, and sensorineural hearing loss, are frequently observed [3]. The overlap of symptoms with other connective tissue disorders, particularly Ehlers–Danlos Syndrome (EDS), often results in misdiagnosis, delaying appropriate intervention and management [7].

### 3.1. Clinical and Genetic Diagnosis

A definitive diagnosis of BCS requires a combination of clinical evaluation and genetic testing [3,4]. Corneal topography in affected individuals typically reveals a diffusely thinned cornea, with a central corneal thickness (CCT) often measuring below 400 µm and, in some cases, even less than 300 µm [3]. Although corneal thinning is present in other connective tissue disorders, the severity and extent of thinning in BCS are notably more pronounced [3]. Additionally, keratoglobus, high myopia, and irregular astigmatism are common findings [2,3]. The extreme fragility of the cornea predisposes individuals to spontaneous rupture or rupture following minor trauma, making early recognition and diagnosis critical [8].

Genetic confirmation plays a pivotal role in differentiating BCS from other similar disorders [3,4,5,6,7,10]. Mutations in the ZNF469 gene (Type 1 BCS) and PRDM5 gene (Type 2 BCS) have been identified as the underlying genetic causes [3,4,6,9,10]. Whole-exome sequencing (WES) has proven to be an effective tool in identifying pathogenic variants, providing diagnostic certainty and guiding clinical management [4]. Early genetic testing is essential for proper diagnosis, family counseling, and preventive strategies to mitigate the risk of severe ocular complications [3,9]. The c.974delG (p.Cys325LeufsX2) variation in PRDM5 is linked to typical ocular manifestations, including severe corneal thinning and keratoglobus. In our patients, the phenotype aligned with other descriptions of PRDM5-associated BCS, which often exhibit more ocular-specific characteristics compared to ZNF469 mutations that may manifest with wider systemic implications.

### 3.2. Challenges in Management and Treatment

Due to the fragile nature of the cornea and sclera in BCS, management is complex and requires a multidisciplinary approach involving ophthalmologists, geneticists, and corneal surgeons [3,9]. There is currently no pharmacological treatment available to halt the progression of corneal thinning, making surgical intervention the primary treatment option for visual rehabilitation [3,9], although few studies reported the use of corneal cross-linking in BCS patients with promising results: Two pediatric patients with brittle cornea syndrome, each with central corneal thickness < 280 μm, underwent transepithelial (epithelium-on) corneal cross-linking with UV fluence scaled to corneal thickness. Both cases achieved better visual acuity and maintained stable endothelial cell counts, indicating that, with protocol modifications, cross-linking may be a viable treatment even in ultra-thin corneas normally excluded by the standard Dresden criteria [11,12,13]. In these two cases, conventional cross-linking was not considered due to contraindications for corneal thickness less than 200 µm. DALK protects the endothelium and potentially decreases rejection risk; nonetheless, its practicality is constrained by significant stromal fragility. In Case 2, patient DALK was unsuccessful owing to intraoperative perforation, requiring a transition to PKP. Nevertheless, it continues to be a viable first-line choice for certain patients with sufficient stromal thickness and intact Descemet’s membrane.

### 3.3. Penetrating Keratoplasty (PK)

Penetrating keratoplasty (PK) is often the treatment of choice in patients with severe corneal thinning or rupture [2,3,9,14]. The PKP was conducted under general anesthesia with a conventional 8.0 mm trephine. The donor graft was affixed using 16 interrupted 10-0 nylon sutures. The anterior chamber was reconstituted with balanced salt solution, and viscoelastic was employed to safeguard the endothelium. Sutures were selectively excised postoperatively after 12 months, contingent upon topographic astigmatism. However, PK in BCS poses significant challenges due to the extreme fragility of the ocular tissues. The need for large grafts, difficulties in suturing, and the high risk of postoperative complications, such as graft rejection, secondary glaucoma, and suture-related infections, make surgical outcomes highly variable [2,3,5,9]. Despite these challenges, PK has been shown to improve visual acuity significantly in affected patients [2,3,9,13]. Long-term graft survival depends on meticulous postoperative care and regular follow-up to monitor for potential complications [3,9]: both patients had outstanding surgical recovery. No significant problems, including graft rejection, glaucoma, or endothelial failure, were noted over the 7-year follow-up period.

### 3.4. Protective Strategies and Lifestyle Modifications

Given the high risk of corneal rupture, protective lifestyle measures are crucial in BCS management [3,8]. Patients are advised to wear protective eyewear to prevent trauma and avoid activities that may increase the risk of ocular injury [3]. Regular monitoring with slit-lamp examination, pachymetry, and anterior segment optical coherence tomography (AS-OCT) is recommended to track disease progression and intervene at the earliest signs of corneal impairment [3].

### 3.5. Multidisciplinary Management

Beyond its hallmark ocular fragility, brittle cornea syndrome (BCS) manifests as a multisystem connective tissue disorder with significant musculoskeletal, dermatologic, audiologic, and other systemic features (joint hypermobility, sensorineural hearing loss, scoliosis, hip dysplasia, cardiac valvular anomalies, aneurysms, dissections, etc.) [15,16,17,18,19,20,21]. These characteristics frequently coincide with other connective tissue illnesses, such as Ehlers–Danlos Syndrome, and may enhance clinical suspicion. Nonetheless, due to the infrequency of BCS and its predominant ocular manifestation, visual characteristics are essential for diagnosis and treatment. To date, there is no disease-specific treatment, so therapy remains supportive and focused on preventing complications. Early diagnosis and personalized multidisciplinary care are critical, as they significantly improve quality of life in BCS patients.

### 3.6. Future Directions in Research and Therapy

Further research is needed to elucidate the molecular mechanisms underlying BCS and explore potential gene-targeted therapies. Advances in genetic medicine, including CRISPR-based genome editing and RNA interference (RNAi), hold promise in the development of future treatments aimed at stabilizing or reversing corneal thinning [22]. Longitudinal studies assessing the long-term outcomes of keratoplasty in BCS patients will provide valuable insights into the durability and effectiveness of surgical interventions [23]. Beyond traditional perforating keratoplasty, Rafat et al. developed a promising cell-free corneal implant—BPCDX—made from medical-grade porcine collagen that is chemically and photochemically double-crosslinked into a transparent, load-bearing hydrogel. A 280 µm lenticule is folded and inserted through a 2–3 mm intrastromal pocket created with a femtosecond laser or manual dissection, eliminating sutures and leaving the native epithelium and endothelium intact. The implant reliably thickened the cornea, flattened maximum keratometry by up to 18 D, and improved best-corrected visual acuity. Outcomes remained stable without rejection or serious complications over 24 months, offering a donor-independent, minimally invasive alternative to keratoplasty in BCS patients [24].

An additional therapeutic frontier aimed at mitigating corneal weakening is intrastromal stem-cell injection: this technique consists of the intrastromal injection of mesenchymal adipose-derived and bone marrow-derived stem cells through a lamellar dissection of the cornea made by a femtosecond laser. Patients with BCS could benefit from these approaches, postponing penetrating keratoplasty for as long as possible, although, so far, they have been used only in keratoconus-affected patients [25,26].

## 4. Conclusions

Brittle cornea syndrome (BCS) is a rare yet profoundly disabling condition that necessitates early recognition and timely intervention to optimize visual outcomes and prevent severe complications, such as spontaneous globe rupture [8]. Given its progressive nature and the high risk of corneal perforation even after minor trauma, prompt diagnosis is essential for initiating appropriate management strategies [2,3]. Early genetic diagnosis plays a crucial role, not only in confirming the condition but also in enabling precise classification, facilitating genetic counseling and guiding individualized treatment approaches [3]. Identifying pathogenic variants through whole-exome sequencing (WES) provides valuable insights into disease mechanisms and potential therapeutic targets [4,10].

Penetrating keratoplasty (PK) has demonstrated efficacy as a viable surgical option for visual rehabilitation in patients with BCS, despite the inherent technical challenges posed by extreme corneal thinning and fragility [3,9]. While PK can restore corneal integrity and improve visual function, its long-term success is highly dependent on meticulous postoperative care. This includes stringent monitoring for complications, such as graft rejection, endothelial cell loss, irregular astigmatism, and secondary glaucoma—conditions that can significantly impact visual prognosis [27,28]. In addition, patient education and ocular protection remain fundamental aspects of long-term management. Given the susceptibility of the cornea to rupture, patients must be advised to take protective measures, including wearing polycarbonate protective eyewear and avoiding activities that pose the risk of ocular trauma [1,6,8].

A multidisciplinary approach is essential for the comprehensive management of BCS. Collaboration among ophthalmologists, corneal surgeons, geneticists, and specialized rehabilitation teams ensures a holistic treatment plan tailored to each patient’s needs [3,7]. Genetic counseling should be offered to affected individuals and their families to discuss inheritance patterns, recurrence risks, and potential preventive measures [3,6,10]. Looking ahead, advancements in genetic research may open new therapeutic avenues for BCS. Gene therapy, pharmacological interventions aimed at strengthening corneal collagen, or bioengineered corneal grafts could revolutionize treatment strategies, offering alternatives to conventional surgical approaches [29,30].

Further research is warranted to explore these possibilities and establish standardized protocols for diagnosis, treatment, and long-term follow-up. Ultimately, improving patient outcomes in BCS requires not only technological and surgical advancements but also a deeper understanding of disease pathophysiology and a commitment to long-term patient care through early intervention, personalized management strategies, and continued research efforts.

## Figures and Tables

**Table 1:** Summary of the study.

	Case 1 (Male, 28 Years Old)	Case 2 (Female, 25 Years Old)
**Main Symptoms**	Progressive visual deterioration, recurrent corneal erosions	Progressive visual deterioration
**Minimum Corneal** **Thickness**	189 µm	157 µm
**Type of Keratoplasty**	Penetrating keratoplasty (PKP)	Deep anterior lamellar keratoplasty (DALK), later converted to PKP
**Reason for Conversion to PKP**	Not applicable	Severe central endothelial perforation
**Postoperative Therapy**	Immunosuppressive	Immunosuppressive
**Preoperative BCVA**	20/200	20/200
**Postoperative BCVA**	20/30	20/25
**Observed Complications**	None after 7 years of follow-up	None after 7 years of follow-up
**Genetic Findings**	PRDM5 gene mutation	PRDM5 gene mutation
**Conclusions**	Continuous monitoring is necessary to prevent complications	DALK can reduce complications when feasible

## Data Availability

Raw data can be provided upon request.
